# Use of artificial intelligence tools in the publishing process: expectations from publishers through author guidelines

**DOI:** 10.3389/frma.2026.1740510

**Published:** 2026-05-14

**Authors:** Josiline Chigwada, Patrick Ngulube

**Affiliations:** School of Interdisciplinary Research and Graduate Studies, University of South Africa, Pretoria, South Africa

**Keywords:** artificial intelligence tools, author guidelines, cope, digital publishing, scholarly communication

## Abstract

**Introduction:**

The rapid integration of artificial intelligence (AI) into scholarly publishing has prompted publishers to develop policies that guide its ethical and practical use. This study explored the question, “What do publishers expect on the use of AI as portrayed in the author guidelines and AI publisher policies?” The investigation was guided by internationally recognized frameworks, including the STM ethical and practical guidelines on AI use, the Committee on Publication Ethics, the World Association of Medical Editors (WAME) recommendations on chatbots and generative AI in scholarly publishing, and the International Committee of Medical Journal Editors (ICMJE) standards.

**Methods:**

A qualitative research approach was employed, drawing from AI-related guidelines and policies from major scholarly publishers. Four publishers were purposively selected based on their global reach, relevance across multiple disciplines, and established AI policies. Data collection was conducted through document and web content analysis, which systematically extracted relevant information from publishers' websites.

**Results:**

A qualitative research approach was employed, drawing from AI-related guidelines and policies from major scholarly publishers. Four publishers were purposively selected based on their global reach, relevance across multiple disciplines, and established AI policies. Data collection was conducted through document and web content analysis, which systematically extracted relevant information from publishers' websites. The findings revealed that AI policies are not standardized across publishers; instead, each adopts its own unique rules regarding AI-assisted writing, authorship, peer review, and disclosure.

**Discussion:**

While prior studies have documented the prevalence of disclosure requirements and authorship prohibitions, the present study moves beyond inventory to critically examine how international frameworks are reflected, adapted, and contested within individual publisher policies, and to surface tensions around enforceability, equity, and disciplinary variability that remain underexplored in existing literature. Consequently, researchers are required to carefully consult the specific policies of their target publishers before submission. The study recommends that all stakeholders in the publishing process be informed about AI-related policies relevant to their roles. Authors should ensure that AI outputs are accurate, reviewers must remain vigilant about unauthorized AI use, and publishers should proactively communicate best practices to maintain integrity and transparency in the scholarly publishing process.

## 1 Introduction

The rapid integration of generative artificial intelligence (AI) tools, particularly large language models (LLMs), into scholarly publishing has fundamentally altered how manuscripts are prepared (Madden et al., 2024; Moulaison-Sandy, 2025; Pongrac, 2024). This transformation has prompted leading international editorial bodies to develop normative frameworks governing acceptable AI use. The International Committee of Medical Journal Editors (ICMJE) updated its recommendations in April 2025 to require explicit disclosure of AI use and to confirm that AI tools cannot qualify as authors because they cannot assume responsibility for published work (International Committee of Medical Journal Editors, 2025). Similar positions are echoed by the Committee on Publication Ethics (COPE, 2023) and the World Association of Medical Editors (WAME). Collectively, these statements set important norms whereby AI may assist, but it cannot assume authorship or accountability, and its use must be explicitly disclosed (World Association of Medical Editors, 2023). At the level of individual publishers, however, policy details vary. Elsevier, for instance, requires authors to disclose any use of generative AI or AI-assisted tools and provides guidance that extends from scientific writing to figures and images (Elsevier, 2025). Springer Nature prohibits the publication of generative AI images and asks peer reviewers not to upload manuscripts into generative AI tools (Springer Nature, 2023). Wiley instructs authors to document and disclose any AI technology used and to verify AI-assisted outputs (Wiley, 2025). These differences reflect both the speed of technological change and distinct risk assessments regarding research integrity, copyright, privacy, and misuse. Despite this emerging guidance, the evidence base on what publishers expect from authors remains uneven and fragmented across disciplines. Bibliometric analysis of publisher instructions found that while the majority disallowed AI as an author and called for disclosure, actual guidance was inconsistent in scope, location, and required detail, suggesting burdens on authors and editors and a risk of policy confusion (Ganjavi et al., 2023, 2024). These publisher-specific variations signal that there is no unified standard governing how AI should be used, disclosed, or governed at the point of submission (Ganjavi et al., 2024).

Existing position statements (e.g., ICMJE, COPE, WAME) are normative and cross-disciplinary, but they do not capture the on-the-ground publisher-specific expectations communicated in author guidelines and policy pages. Furthermore, many publisher policies are living documents disseminated across multiple sites, such as author services, web pages, and editorial policies, making systematic comparison difficult. There is, therefore, a need for an up-to-date, comparative synthesis that maps how publishers describe acceptable AI use across the research and publishing workflow, what benefits they themselves highlight, and what risks and compliance requirements they emphasize so that authors, editors, and research administrators can align practice to policy with clarity. It is against this background that the study sought to answer the following research question: What do publishers expect regarding the use of AI, as portrayed in author guidelines and AI publisher policies? To address this gap, the study is guided by four internationally recognized frameworks, the STM Ethical and Practical Guidelines on AI use (STM, 2023), the COPE position statement on authorship and AI tools, the WAME recommendations on chatbots and generative AI in scholarly publishing, and the ICMJE standards. These frameworks collectively informed the data extraction protocol, coding categories, and interpretive lens applied throughout the study, enabling a systematic evaluation of how individual publisher policies align with, extend, or deviate from established international norms. The analytical contribution of this study lies in moving beyond a descriptive inventory of policies to a critical, framework-informed synthesis that identifies convergences, divergences, tensions, and practical implications for key stakeholders in the scholarly publishing ecosystem.

## 2 Conceptual framework

The study was guided by the STM ethical and practical guidelines on AI use, COPE position statement on authorship and AI tools, WAME recommendations on chatbots and generative artificial intelligence in relation to scholarly publications, and the ICMJE. These will be matched against the policies and AI author guidelines provided by publishers on their websites to pinpoint what publishers expect during the publishing process.

### 2.1 STM ethical and practical guidelines on AI use

These guidelines showcase the recommendations for best practices in the use of GenAI in the publication process. They assist publishers in developing policies for all the stakeholders in the publishing process on how to use AI (STM, 2023). In preparing a manuscript, the following principles are recommended:

a) “Using publicly available GenAI as a basic tool that supports authors in refining, correcting, formatting, and editing texts and documents is permissible.
b) Authors must disclose any use of GenAI that transcends those use cases so an editorial decision can be made as to its legitimacy.
c) GenAI cannot be used to create, alter, or manipulate original research data and results, such as images, blots, photographs, x-rays, and measurements.
d) GenAI cannot be credited as an author of a published work” (STM, 2023).

### 2.2 Committee on Publication Ethics (COPE) position statement on authorship and AI tools

COPE acknowledges that the use of AI tools in research publications is growing, but these tools cannot be listed as authors (COPE, 2023). This is because AI tools cannot meet the requirements of authorship, as they are not responsible for any work submitted for publication. Among other things, AI tools cannot manage copyright and licensing agreements and cannot handle conflicts of interest. Therefore, it is recommended that authors who utilize AI should disclose in the materials and methods section how they have used AI and indicate which tools were used. This can include writing the manuscript, creating images or other graphs, or collecting and analyzing data. This is because authors are responsible for what is included in the manuscript, including content generated by AI tools, and should also address issues of publishing ethics.

### 2.3 World Association of Medical Editors (WAME) recommendations on chatbots and generative artificial intelligence in relation to scholarly publications

The statement reflects the proliferation of chatbots and their expanding use in scholarly publishing, as well as emerging concerns about the authenticity of content generated by chatbots. The recommendations acknowledge that chatbots are used for various functions in scholarly publishing. These can be simple word-processing tasks, the generation of ideas and text, and substantive research. The recommendations are intended to inform editors and help them develop policies for the use of chatbots in papers published by these publishers. They aim to help authors and reviewers understand how best to attribute the use of chatbots in their work, and to address the need for all editors to have access to manuscript screening tools. The recommendations are:

1. Only humans can be authors, and chatbots cannot be authors. The author must be a legal person and chatbots do not meet the International Committee of Medical Journal Editors authorship criteria of being able to give final approval of the version to be published, and to be accountable for all aspects of the work in ensuring that questions related to the accuracy and integrity of any part of the work are appropriately investigated and resolved (International Committee of Medical Journal Editors, 2025). This is because an artificial intelligence tool cannot understand a conflict-of-interest statement and does not have the legal standing to sign a statement. They do not have an independent affiliation with their developers. Since authors submitting a manuscript must ensure that all named authors meet authorship criteria, chatbots cannot be included as authors (World Association of Medical Editors, 2023).

2. Authors should acknowledge the sources of their materials, i.e., authors should be transparent when chatbots are used and provide information about how they were used. Authors should indicate the extent and type of use of chatbot publications. This aligns with the ICMJE recommendation to acknowledge writing assistance (International Committee of Medical Journal Editors, 2025) and to provide detailed information about how the study was conducted and the results generated (International Committee of Medical Journal Editors, 2025). It was noted that if AI is used to generate new text, such use should be in the acknowledgment section, and all prompts used to generate new text should be specified. In addition, if AI is used to help report results, generate analytical work, or write computer codes, it should be stated in the body of the paper, in both the abstract and the methods section. The full prompt used to generate the research results, the time, and date of query, the AI tool used, and its version should be provided (World Association of Medical Editors, 2023).

3. Authors must take public responsibility for their work since authors are responsible for the material provided by a chatbot in their paper, including the accuracy of what is presented and the absence of plagiarism. It is the author's responsibility to ensure that the content reflects the author's data and ideas and is not plagiarized, fabricated, or falsified. They should specify what they have done to mitigate the risk of plagiarism, provide a balanced view, and ensure the accuracy of all their references (World Association of Medical Editors, 2023).

4. Editors and reviewers should specify, to authors and each other, any use of chatbots in the evaluation of the manuscript and generation of reviews and correspondence. If they use chatbots in their communications with authors and each other, they should explain how they were used. Editors and reviewers should be aware that chatbots retain the prompts fed to them, including manuscript content, and supplying an author's manuscript to a chatbot breaches the confidentiality of the submitted manuscript (World Association of Medical Editors, 2023).

5. Editors need appropriate digital tools to deal with the effects of chatbots on publishing and help them detect content generated or altered by AI. Such tools should be made available to editors regardless of their ability to pay for them, for the good of science and the public, and to help ensure the integrity of healthcare information and reduce the risk of adverse health outcomes. Editors need access to tools that will help them evaluate content efficiently and accurately. This is particularly important for medical publishers, where the adverse consequences of misinformation include potential harm to people (World Association of Medical Editors, 2023).

### 2.4 International Committee of Medical Journal Editors – ICMJE

ICMJE developed the recommendations to review best practices and ethical standards in the conduct and reporting of research and other materials published by medical publishers and to help authors, editors, and others involved in peer review and biomedical publishing create and distribute accurate, clear, reproducible, unbiased medical articles. The recommendations may also provide useful insights into the medical editing and publishing process for the media, patients and their families, and general readers (International Committee of Medical Journal Editors, 2025). The ICMJE helps in defining the roles of authors and contributors, and recommends that authorship be based on the following 4 criteria:

1. “Substantial contributions to the conception or design of the work; or the acquisition, analysis, or interpretation of data for the work; AND

2. Drafting the work or reviewing it critically for important intellectual content; AND

3. Final approval of the version to be published; AND

4. Agreement to be accountable for all aspects of the work in ensuring that questions related to the accuracy or integrity of any part of the work are appropriately investigated and resolved” (International Committee of Medical Journal Editors, 2025).

It was stated that activities that do not qualify one to be an author include acquisition of funding; general supervision of a research group or general administrative support; and writing assistance, technical editing, language editing, and proofreading (International Committee of Medical Journal Editors, 2025). Therefore, these contributors should be acknowledged, and the use of AI for writing assistance should be reported in the acknowledgment section. In addition, chatbots should not be listed as authors since they are not responsible for the articles' originality, integrity, and accuracy, which are requirements for authorship. This means that only humans can be attributed as authors, as they are responsible for the manuscript. Therefore, authors should review and edit their manuscripts before submission, as AI can generate incorrect or biased information. It is further noted that authors should not cite AI nor list it as a co-author and should avoid any form of plagiarism.

## 3 Research methodology

This study adopted a qualitative research design, which is appropriate for exploring meanings, expectations, and interpretations rather than measuring frequencies or testing hypotheses (Lim, 2024). Since the research question seeks to understand publishers' perspectives and positions as expressed in textual documents, qualitative inquiry provides the most suitable framework. Qualitative methods allow for in-depth examination of the content, language, and framing of policy statements to capture the tones that may be lost in quantitative approaches (Creswell and Poth, 2024; Given, 2016). The population for this study comprised AI-related guidelines and policies from major scholarly publishers, which are Emerald, Frontiers, Sage, and Taylor and Francis. From this population, four publishers were purposively selected, and the rationale for selecting these four is threefold. Global influence and market dominance show that these publishers represent a significant proportion of global scholarly output (Larivière et al., 2015). Their policies, therefore, carry considerable weight in shaping norms and practices across disciplines. In addition, the diversity of subject coverage ensures that each of these publishers covers a wide range of disciplines, from the sciences and medicine to the social sciences and humanities, thereby enabling the analysis to capture cross-disciplinary perspectives on AI policy. Moreover, policy leadership shows that these publishers have been among the first to articulate detailed policies regarding generative AI use, authorship, and disclosure, making them appropriate cases for a study examining current expectations. This purposive sampling approach is suitable in qualitative research, where the goal is to select information-rich cases that best address the research question (Patton, 2015). Inclusion and exclusion criteria were applied to ensure analytical rigor and consistency. Publishers were included if they maintained a publicly accessible AI-specific policy or had explicit AI-related clauses embedded in their author guidelines; operated at a global scale with multi-disciplinary coverage; and had updated their AI-related guidance within the preceding 2 years to reflect the generative AI developments since 2022. Publishers whose AI guidance was limited to a single brief statement lacking operational detail, or whose policies could not be accessed in English, were excluded. The unit of analysis was the individual policy document or web page, encompassing author guidelines, instructions to authors, editorial policy statements, and supplementary AI guidance documents published on each publisher's official website. Data collection was conducted between September and December 2025, a period during which all four publishers‘ AI policies were actively maintained and publicly accessible. Given the rapidly evolving nature of AI governance in publishing, the data collection window is explicitly reported to enable readers to contextualize the currency of the findings and to facilitate future comparative studies.

Data was collected using web content analysis of author guidelines and AI-related policy statements published on the official websites of the selected publishers. Web content analysis is a systematic technique for gathering and analyzing publicly available online documents to understand how organizations present and communicate issues of interest (Krippendorff, 2022). The justification for using web content analysis includes authenticity and accessibility, where publishers communicate their official positions to authors and reviewers through web-based platforms, making their websites the most reliable source for current, authoritative policy documents. The other one is the dynamic nature of AI policy since policies on AI are continuously updated in response to technological advances, and web content analysis allows for capturing the most up-to-date positions as they are formally presented online (Neuendorf, 2017). Finally, suitability for textual analysis is enhanced when web content analysis allows for systematic coding and interpretation of textual data, aligning with the qualitative approach of identifying themes, expectations, and patterns in publishers' communication.

The study utilized a document analysis protocol as the main data collection instrument. The protocol guided the extraction of relevant sections from the publishers' websites, including author guidelines, instructions to authors, and editorial policies; specific statements on AI-assisted writing, authorship, peer review, and disclosure requirements; supplementary policy documents or blog posts where publishers provided clarifications. The protocol included categories such as permitted AI uses, prohibited AI uses, disclosure requirements, authorship rules, treatment of AI-generated images/data, peer review guidance, and implementation mechanisms. This ensured systematic and consistent data extraction across all four publishers. Document analysis is well established as an instrument for collecting and interpreting qualitative data, particularly where organizational policies and guidelines are the focus (Bowen, 2009). The extracted texts were analyzed using thematic analysis, which involved coding, categorizing, and identifying recurring themes across the four publishers' documents. Following Braun and Clarke (2006) framework, the analysis proceeded through stages of familiarization, coding, theme generation, and interpretation. This approach allowed for comparison of commonalities and divergences in publishers' expectations regarding AI use.

## 4 Findings

The findings showed that AI policies differ from one publisher to another, and they are not standardized. Every publisher has its own unique rules, and the researchers should read the policies and guidelines before submission. These themes emerged consistently across the four publisher policy documents and align directly with the dimensions of the conceptual framework outlined in the preceding section. Where convergence exists across publishers, it was noted; where divergence or tensions appear, they were highlighted and critically examined.

### 4.1 AI authorship and accountability

One of the most consistent findings across all four publishers is the unambiguous prohibition of listing AI tools as authors. This convergence is total and unreserved, with all publishers stating explicitly that AI bots and generative AI systems cannot be credited as authors. Frontiers states that AI tools must not be listed as authors, while Sage guidelines note that AI tools cannot be recognized as a co-author. Emerald grounds this prohibition in authorship criteria, specifying that using generative AI to generate an abstract or literature review is not permissible precisely because it would violate those criteria. The alignment across publishers reflects the influence of the STM, COPE, WAME, and ICMJE frameworks, all of which define authorship as requiring human accountability, the ability to take responsibility for the work, and the capacity to manage legal and ethical obligations, which cannot be fulfilled by AI tools and applications.

Beyond the authorship prohibition, all four publishers assign full accountability for AI-assisted content to the human author. Frontiers specifies that authors are accountable for the originality, validity, and integrity of their submissions, and that they must review AI-generated content and confirm its accuracy. Sage reinforces this by stating that authors are responsible for the submitted work and must verify the accuracy of any AI-generated content, correcting any errors. Taylor and Francis, while more permissive in tone, embraces AI as an opportunity for generating ideas and supporting non-native English-speaking authors, but this permissiveness does not extend to relieving authors of the responsibility of what is ultimately submitted. This cross-publisher census on human accountability is significant since it places the burden of quality assurance, ethical compliance, and integrity squarely on the author, regardless of how much AI assistance was used in the process.

### 4.2 Disclosure requirements and transparency

Disclosure of AI use is a second area of broad convergence, though the specifics of what must be disclosed and where vary across publishers. Frontiers and Sage both mandate explicit disclosure when generative AI tools are used to generate text, images, or references. Frontiers requires the author to clearly acknowledge the use of any AI tool, while Sage states that authors must indicate the use of AI in the manuscript and clearly reveal AI-generated content along with the submission. Sage further distinguishes between assistive AI tools such as Grammarly and language improvement software, and generative AI tools such as ChatGPT or DALL-E, noting that only the latter category requires mandatory disclosure. This assistive/generative distinction is a notable policy distinction as it acknowledges that not all AI use is equivalent and attempts to calibrate disclosure obligations accordingly. Emerald takes a more granular approach to transparency since it requires all AI-created images to be flagged in the text and cited, and that in-text reporting of statistics using AI be explicitly prohibited, though AI may be used to aid analysis, provided this is disclosed. This layered approach reflects a sophisticated attempt to distinguish between different functional uses of AI within a single manuscript. Frontiers extends transparency obligations to editors and peer reviewers, explicitly stating that they must not upload files, images, or information from an unpublished manuscript into AI tools, a provision that addresses confidentiality risks as much as transparency. Across all four publishers, the principle of transparency serves as a foundational governance mechanism, positioning disclosure not merely as a procedural requirement but as an ethical obligation that preserves the integrity of the scholarly record.

### 4.3 Permitted and prohibited use across the publishing workflow

The area of greatest divergence across publishers lies in what AI uses are permitted or prohibited across different stages of the publishing workflow. There is, however, consistent agreement on a small set of absolute prohibitions. All four publishers prohibit the use of AI to artificially create or modify core research data. Sage explicitly states this, and the prohibition is implied in the data integrity requirements of Emerald and Frontiers. Similarly, all four publishers prohibit editors and reviewers from sharing submitted manuscripts or peer review reports with publicly available AI tools, citing confidentiality, data security, and the risk of intellectual property leakage. Emerald adds that sensitive personal information must not be shared on AI platforms, since such information is collected for commercial purposes by AI providers. In contrast, language editing and readability improvement represent the most universally accepted AI use across all four publishers, Emerald permits copyediting using AI to improve language and readability, Frontiers supports responsible use that includes language improvement provided confidentiality and copyright protections are respected, Sage distinguishes assistive language tools as exempt from mandatory disclosure, and Taylor and Francis specifically highlights AI's value in supporting authors who do not use English as their first language. This convergence on language assistance reflects a recognition that AI-powered language tools are now deeply embedded in academic writing practice globally.

Image generation shows the sharpest divergence among the four publishers. Emerald permits the submission and publication of AI-created images but requires that they be flagged and cited. Sage permits AI-generated images with disclosure, while Frontiers prohibits them outright in some of its journals. This inconsistency means that authors working across different publishers face genuinely contradictory expectations regarding the same practice. Literature review assistance is similarly contested, where Emerald explicitly prohibits AI use in generating literature reviews because this constitutes a core intellectual contribution, while Taylor and Francis takes a more permissive view. Data analysis occupies a middle ground where it is conditionally permitted with disclosure across all four publishers, while in-text reporting of AI-generated statistics is explicitly prohibited by Emerald. Table 2 summarizes the main activities that are allowed or restricted across the four publisher policies.

### 4.4 Enforcement mechanisms and compliance expectations

All four publishers articulate compliance expectations, but enforcement mechanisms remain notably vague across the board. Sage is the most explicit about consequences, stating that undisclosed use of generative AI tools would lead publishers to take appropriate corrective action. The policies across Taylor and Francis, Emerald, and Frontiers describe expected author conduct in detail but similarly stop short of specifying penalties for non-compliance, detection procedures, or escalation pathways. This gap between normative expectations and operational enforcement is a structural weakness in the current AI governance landscape as represented by these four publishers. In terms of compliance expectations placed on authors, Sage provides the most comprehensive guidance, requiring authors to verify AI-generated content for accuracy and hallucinations. Authors are also encouraged to check sources and citations, including those generated by AI, cite AI-generated content appropriately, avoid plagiarism and copyright infringement, review content for AI biases, acknowledge AI limitations, check specific submission guidelines, and remain informed about ongoing debates regarding AI-generated content. This extensive checklist effectively transfers substantial editorial quality-control responsibility onto authors. Frontiers reinforces the compliance expectation by requiring human oversight and transparency at all stages of AI use, and further notes that some individual journals within its portfolio may restrict AI use more tightly than the general publisher policy. This provision further complicates compliance for authors submitting across that publisher's portfolio. It was further observed that AI tools can be used to create fabricated submissions and to subvert peer review for commercial gain, and that current language models are not yet fully objective or factual, highlighting why robust compliance mechanisms are essential. Table 1 summarizes the main activities that are allowed by different publishers.

**Table 1:** Publisher policies on AI use.

Activity	Policies on AI use	Implications
Idea and hypothesis formation COPE (2023); Hosseini et al. (2025); Lin (2024)	Mostly forbidden since it is considered a core intellectual contribution requiring human originality and accountability.	Researchers must independently generate research ideas; AI cannot be credited with intellectual ownership.
Writing assistance Cleland et al. (2026); Elsevier (2025); Resnik and Hosseini (2026)	Accepted with proper disclosure. Must acknowledge AI tools used. AI is viewed as a support tool for language refinement rather than content generation.	Authors must disclose AI use and ensure responsibility for the accuracy and integrity of content.
Literature review assistance Chelli et al. (2024); Emerald Publishing (2024); Sun et al. (2024)	Mixed policies depending on the publisher. Some allow with disclosure, and others prohibit due to concerns about bias and hallucination.	Researchers should verify AI-generated summaries and comply with specific publisher guidelines.
Image and figure creation Ganjavi et al. (2024); Chauhan and Currie (2024); Sage (2025)	Varies by publisher and author should check specific guidelines before use.	Authors must confirm originality and may need to provide methodological transparency.
Data analysis and interpretation Alduais et al. (2025); Arar et al. (2025); Resnik and Hosseini (2026)	Restricted in most publishers. Requires explicit disclosure and justification.	Disclosure and methodological justification are required when AI tools are used.
Peer review process Doskaliuk et al. (2025); Kemal (2025); Mollaki (2024)	Not allowed by most publishers, and reviewers cannot be reengaged if discovered.	Reviewers must not use AI tools; breaches may lead to disqualification or sanctions.

## 5 Discussion

The conceptual framework of this study was grounded on ethical and practical guidelines developed by international publishing and editorial bodies, including STM (2023); COPE (2023); World Association of Medical Editors (2023), and International Committee of Medical Journal Editors, 2025. These guidelines provide the normative basis for acceptable and ethical use of generative artificial intelligence (GenAI) in scholarly publishing, particularly emphasizing disclosure, transparency, human accountability, and the prohibition of AI as an author. The findings of this study show how these global frameworks are reflected, adapted, or contested within individual publisher policies, highlighting both convergence and divergence in practice. The STM guidelines emphasize that while AI can be a supportive tool for refining, correcting, and editing manuscripts, its use should not extend to the manipulation of research data or attribution of authorship. This principle was echoed in the findings, where most publishers prohibited AI use in generating core research outputs, such as results or statistical analysis, but permitted its use for language editing and readability (Taylor and Francis, 2025; Sage, 2025). Similarly, COPE's position that AI cannot be credited as an author because it lacks accountability (COPE, 2023) was consistently observed across the policies of the four publishers reviewed, where publishers universally stated that AI bots must not be listed as authors. This reflects a growing consensus in publishing ethics that aligns with the conceptual framework's stipulation of human responsibility for all published outputs (Shamoo and David, 2022).

However, the findings also reveal variations in how publishers operationalize these principles. For example, Taylor and Francis highlighted the opportunities of AI in idea generation and overcoming language barriers, a more permissive stance compared to Emerald, which strictly prohibited AI-generated abstracts and literature reviews, as shown in Table 2. These differences highlight that while the conceptual framework provides general ethical anchors, the interpretation at the publisher level depends on disciplinary priorities and publisher philosophies. WAME (2023) emphasis on authenticity and the risk of misinformation resonates with Emerald's restrictive policies, while Sage's more flexible guidelines reflect the balance between innovation and safeguarding research integrity. This tension between enabling technological innovation and maintaining ethical safeguards has been noted in recent scholarship on AI in publishing (Clark, 2025; Pongrac, 2024). Beyond mapping convergences, it is necessary to critically examine the feasibility and enforceability of the policies identified. While all four publishers require disclosure of AI use, none of the reviewed policies articulate robust verification mechanisms to confirm that disclosure requirements are being met. The reliance on author self-reporting, without independent verification or standardized auditing, raises legitimate questions about compliance in practice (Ganjavi et al., 2024). Publishers generally describe corrective action only in vague terms such as appropriate measures, leaving significant ambiguity about what consequences follow a policy breach. The gap between policy articulation and enforcement infrastructure represents a meaningful limitation of the current governance landscape that warrants further attention from publishing associations and institutional policymakers.

**Table 2:** Comparative analysis of publisher AI policies.

Policy dimension	Taylor and Francis	Emerald	Frontiers	Sage	Comparative insight
AI as author	No	No	No	No	Universal consensus that AI lacks accountability and cannot hold authorship responsibility.
**Disclosure mandatory**	Yes	Yes	Yes	Yes	Strong alignment across publishers, indicating transparency as a foundational principle.
**Literature review**	Conditional	Conditional	Conditional	Conditional and should be disclosed	Variation reflects differing tolerance levels for AI-assisted synthesis of knowledge.
**Image generation**	Conditional	Conditional	Prohibited	Conditional	Increasing caution due to risks of fabricated or misleading visual outputs.
**Data analysis**	Conditional	Conditional	Conditional	Conditional	Uniform caution suggests recognition of AI's potential, but the need for oversight.
**Peer review use**	Prohibited	Prohibited	Prohibited	Prohibited	Uniformity shows the importance of the restriction on peer review.
**Enforcement**	Described	Described	Described	Described	Publishers emphasize governance structures, though enforcement rigor may vary.

Furthermore, the findings prompt important reflection on equity and power asymmetries. The expectation that authors personally verify all AI-generated content for accuracy, correct hallucinations, and navigate rapidly changing policies across publishing places a disproportionate burden on researchers who may lack institutional support, advanced English proficiency, or familiarity with evolving AI tools. Taylor and Francis's more permissive stance toward AI-assisted language improvement reflects an awareness that non-native English-speaking researchers face distinct challenges. However, this stance sits in tension with the stricter policies of other publishers, creating an uneven compliance landscape. Early-career researchers who may rely more heavily on AI assistance during manuscript preparation are similarly disadvantaged by inconsistent policies and the absence of publisher-provided guidance on navigating policy differences across publishers. These equity considerations remain largely unaddressed in the current policy documents reviewed and represent an important area for future scholarship and publisher reflection.

The conceptual framework also highlights the importance of transparency, requiring that authors disclose the use of AI tools in manuscripts. This principle was echoed in the findings, where Frontiers and Sage mandated disclosure when generative AI was used to produce text, images, or references. The requirement for authors to verify the accuracy of AI-generated content, particularly in Sage's guidelines, aligns with ICMJE's (2025) criteria of authorship, which emphasize accountability and responsibility for integrity and originality. The insistence on disclosure also addresses ongoing concerns about AI “hallucinations,” plagiarism risks, and fabricated references (Stokel-Walker, 2023). Notably, the conceptual framework highlights the risk of breaching confidentiality when editors or reviewers use AI tools. This concern was reflected in the findings, where several publishers explicitly prohibited editors and peer reviewers from uploading manuscripts to AI platforms. This convergence highlights how editorial bodies and publishers are adapting to the new ethical risks posed by AI, particularly around data security, confidentiality, and potential misuse (COPE, 2023; Moulaison-Sandy, 2025; Yousaf, 2025). In sum, the conceptual framework provided by international editorial bodies serves as the ethical compass guiding AI use in scholarly publishing, while the findings demonstrate how these principles are translated into heterogeneous publisher policies. While there is broad agreement on core issues, such as prohibiting AI authorship, requiring disclosure, and maintaining human accountability, differences remain in areas like literature review support, image creation, and idea generation. These divergences reflect the evolving nature of AI governance in scholarly publishing, where global principles are being locally interpreted by publishers. As recent scholarship suggests, ongoing dialogue between editorial associations, publishers, and researchers is necessary to harmonize policies while accommodating disciplinary differences (Bender et al., 2021).

## 6 Conclusion and recommendations

The study examined what publishers expect regarding AI use, as articulated in author guidelines and AI-related policies across four major scholarly publishers. The findings confirm that AI governance in scholarly publishing is both active and fragmented. All four publishers examined prohibit listing AI as an author and require human accountability for AI-assisted content, yet they diverge considerably in what they permit, how they frame disclosure obligations, and how they address enforcement. These findings are grounded in the documentary analysis of publicly available publisher policies and should be understood as a mapping of stated expectations, not as evidence of actual author behavior, compliance rates, or measurable outcomes. The study does not empirically assess whether these policies reduce policy breaches, enhance trust, or improve compliance in practice. Such claims lie beyond the scope of a qualitative policy document analysis and would require empirical investigation. Across the four publishers examined, the study found that responsible, limited AI assistance, particularly for language editing and readability, is broadly accepted, provided authors retain intellectual control, verify accuracy, and comply with disclosure requirements. Uploading confidential materials such as manuscripts under review to external AI platforms is explicitly prohibited, as is listing AI as an author. Publishers themselves identify potential benefits of AI, including improved accessibility for non-native English-speaking researchers, enhanced editorial screening, and streamlined matching.

Publishers acknowledge substantial risks associated with AI use in scholarly publishing, including factual errors, fabricated references, unattributed text, confidentiality breaches, and copyright concerns, particularly with AI-generated images. The stated benefits and risks are drawn directly from policy documents and represent publishers' own framing of the AI landscape, and they are not independently verified or empirically assessed outcomes of this study. The analytical contribution of this study lies in producing a comparative, framework-informed synthesis of AI governance across major publishers, mapping areas of convergence and divergence in permitted uses, disclosure obligations, authorship rules, image and data provisions, and peer-review safeguards. This synthesis is intended to support authors navigating submission requirements, editors reviewing publisher policies, institutional policymakers crafting researcher guidance, and librarians delivering compliance and research integrity training. Whether greater policy transparency will, in practice, lead to improved compliance, reduced policy breaches, or enhanced trust in the scholarly record remains an open empirical question that this study is not designed to answer, and that future research should address through direct investigation of stakeholder behavior and outcomes. Future studies should investigate the perspectives of authors, editors, and reviewers on AI policies across different publishers, explore how stakeholders are applying these policies in practice, examine the effectiveness of enforcement mechanisms in deterring breaches, and consider longitudinal designs capable of tracking how AI governance in scholarly publishing evolves.

## Data Availability

Publicly available datasets were analyzed in this study. This data can be found here: https://taylorandfrancis.com/our-policies/ai-policy/.
